# Heart Rate and Extracellular Sodium and Potassium Modulation of Gap Junction Mediated Conduction in Guinea Pigs

**DOI:** 10.3389/fphys.2016.00016

**Published:** 2016-02-02

**Authors:** Michael Entz, Sharon A. George, Michael J. Zeitz, Tristan Raisch, James W. Smyth, Steven Poelzing

**Affiliations:** ^1^Department of Biomedical Engineering and Mechanics, Virginia Polytechnic Institute and State UniversityBlacksburg, VA, USA; ^2^Virginia Tech Carilion Research Institute and Center for Heart and Regenerative Medicine, Virginia Polytechnic Institute and State UniversityRoanoke, VA, USA; ^3^Translational Biology, Medicine, and Health, Virginia Polytechnic Institute and State UniversityBlacksburg, VA, USA; ^4^Department of Biological Sciences, College of Science, Virginia Polytechnic Institute and State UniversityBlacksburg, VA, USA

**Keywords:** electrophysiology, cardiac conduction, ephaptic coupling, gap junction

## Abstract

**Background:** Recent studies suggested that cardiac conduction in murine hearts with narrow perinexi and 50% reduced connexin43 (Cx43) expression is more sensitive to relatively physiological changes of extracellular potassium ([K^+^]_o_) and sodium ([Na^+^]_o_).

**Purpose:** Determine whether similar [K^+^]_o_ and [Na^+^]_o_ changes alter conduction velocity (CV) sensitivity to pharmacologic gap junction (GJ) uncoupling in guinea pigs.

**Methods:** [K^+^]_o_ and [Na^+^]_o_ were varied in Langendorff perfused guinea pig ventricles (Solution A: [K^+^]_o_ = 4.56 and [Na^+^]_o_ = 153.3 mM. Solution B: [K^+^]_o_ = 6.95 and [Na^+^]_o_ = 145.5 mM). Gap junctions were inhibited with carbenoxolone (CBX) (15 and 30 μM). Epicardial CV was quantified by optical mapping. Perinexal width was measured with transmission electron microscopy. Total and phosphorylated Cx43 were evaluated by western blotting.

**Results:** Solution composition did not alter CV under control conditions or with 15μM CBX. Decreasing the basic cycle length (BCL) of pacing from 300 to 160 ms decreased CV uniformly with both solutions. At 30 μM CBX, a change in solution did not alter CV either longitudinally or transversely at BCL = 300 ms. However, reducing BCL to 160 ms caused CV to decrease more in hearts perfused with Solution B than A. Solution composition did not alter perinexal width, nor did it change total or phosphorylated serine 368 Cx43 expression. These data suggest that the solution dependent CV changes were independent of altered perinexal width or GJ coupling. Action potential duration was always shorter in hearts perfused with Solution B than A, independent of pacing rate and/or CBX concentration.

**Conclusions:** Increased heart rate and GJ uncoupling can unmask small CV differences caused by changing [K^+^]_o_ and [Na^+^]_o_. These data suggest that modulating extracellular ionic composition may be a novel anti-arrhythmic target in diseases with abnormal GJ coupling, particularly when heart rate cannot be controlled.

## Introduction

Normal cardiac conduction is critical for maintaining efficient pumping of the heart, and abnormal conduction can lead to arrhythmias and sudden cardiac death. Cardiac conduction is dependent on propagation of electrical signals from cell-to-cell. Cell membrane depolarization can occur by raising the intracellular potential via direct axial current through high resistance gap junctions (gap junctional coupling), or by decreasing the extracellular potential between closely adjacent cells (ephaptic coupling) (Kucera et al., 2002; Sperelakis and McConnell, 2002; Mori et al., 2008; Veeraraghavan et al., 2012, 2014). The concept of ephaptic coupling (EpC) is an old theory that has received renewed interest recently. While considered controversial in the cardiac field, EpC is more widely accepted in neurology (Bokil et al., 2001; Anastassiou et al., 2011; Su et al., 2012; Van der Goes van Naters, 2013; Maïna et al., 2015) and has even been proposed to be important for uterine contraction (Young, 2007). An elegant review by Nicholas Sperelakis and Keith McConnell in 2002 summarizes 6 possible mechanisms for EpC (Sperelakis and McConnell, 2002). Despite the many proposed mechanisms, the most prominent computational descriptions of EpC are electric field coupling (Rohr et al., 1998; Sperelakis and McConnell, 2002; Lin and Keener, 2010), or a combination of electric field coupling and the alteration of ionic concentrations within restricted extracellular microdomains (Mori et al., 2008; Hand and Peskin, 2010; Lin and Keener, 2010, 2013, 2014). Importantly, EpC is a theoretical parallel pathway to gap junction (GJ) mediated cell-to-cell electrical coupling.

Recent evidence in guinea pig ventricular myocardium (Veeraraghavan et al., 2012) demonstrates that inducing acute interstitial edema can increases intercellular separation within the perinexal intercalated disc microdomain and slow cardiac conduction by mechanisms consistent with mathematical predictions of EpC (Lin and Keener, 2010, 2013, 2014). Further, increasing perinexal width hypersensitizes myocardial conduction to pharmacologic GJ uncoupling (Veeraraghavan et al., 2012) and sodium channel inhibition (Veeraraghavan et al., 2015). This hypersensitivity is also consistent with computational predictions of EpC. Importantly, the corollary statement is that narrowing intercellular separation within the perinexus should decrease conduction sensitivity to GJ uncoupling and sodium channel inhibition.

In mice, we previously demonstrated that the relationships between cardiac conduction and GJs, perinexal width, and extracellular concentrations of sodium ([Na^+^]_o_) and potassium ([K^+^]_o_) are complex (George et al., 2015). Since it has also been shown that a change in [K^+^]_o_ from 5 to 8 mM increased CV in guinea pig hearts (Kagiyama et al., 1982; Buchanan et al., 1985), one would expect a similar increase in mice. However, in mouse hearts with the native compliment of Cx43 (wild type), and wide perinexi (>15 nm), cardiac conduction was reduced when [K^+^]_o_ was raised from 4.6 to 6.1mM and concurrently [Na^+^]_o_ was decreased from 155 to 147mM. More intriguing was the finding that the wild type hearts were insensitive to the same changes of [K^+^]_o_ and [Na^+^]_o_ when perinexal width was reduced. However, when Cx43 was reduced by 50%, hearts with narrow perinexi perfused with a solution containing the higher [K^+^]_o_ and lower [Na^+^]_o_ had slower conduction relative to the solution with lower [K^+^]_o_ and higher [Na^+^]_o_. Therefore, the width of the perinexus has important effects on modulating cardiac conduction sensitivity to alterations in [K^+^]_o_ and [Na^+^]_o_, and these effects are unmasked by GJ uncoupling.

Importantly, the relationship between conduction and perinexal width does not appear to be species dependent as similar findings were observed in guinea pig (Veeraraghavan et al., 2015). However, it remains unknown whether small extracellular changes in [K^+^]_o_ and [Na^+^]_o_ can alter conduction sensitivity to pharmacologic GJ coupling in guinea pig.

The purpose of this study was twofold. First, we wanted to determine if the observed response of cardiac conduction to [K^+^]_o_ and [Na^+^]_o_ is similar in normal guinea pig hearts as was observed in Cx43 wild type mouse hearts with narrow perinexi. Second, we sought to demonstrate that pharmacologically uncoupling GJs with a non-specific GJ uncoupler like carbenoxolone (CBX) would unmask conduction sensitivity to [K^+^]_o_ and [Na^+^]_o_ in guinea pig hearts with narrow perinexi.

## Materials and methods

This study abides by and follows all guidelines set forth by the Institutional Animal Care and Use Committee at Virginia Polytechnic Institute and State University and NIH *Guide for the Care and Usage of Laboratory Animals*.

### Langendorff perfusion

Male retired breeder Hartley albino guinea pigs (Hilltop, Scottdale, PA, *N* = 28, 800–1200 g, 12–19 months old) were anesthetized using sodium pentobarbital [Nembutal, 30mg/kg IP]. The heart was extracted, retrogradely perfused in a Langendorff perfusion apparatus and the atria excised to reduce competitive stimulation. The hearts were perfused with constant flow to maintain pressure between 40 and 55 mm Hg. Tyrode's solutions were altered as described in Table 1. The laboratory's historical Tyrode's composition in Table 1 is presented as a point of reference for the modified solutions used in these experiments.

**Table 1:** **Modified Tyrode's solution compositions (mM)**.

	**Historical**	**Solution A**	**Solution B**
**GP Tyrode's solution (mmol/L)**			
NaCl	140	147.8	140
NaOH	5.5	5.5	5.5
**Total [Na^+^]**	**145.5**	**153.3**	**145.5**
KCl	4.56	4.56	6.95
**Total [K^+^]**	**4.56**	**4.56**	**6.95**
CaCl_2_	1.25	1.25	1.25
Dextrose	5.5	5.5	5.5
MgCl_2_	0.7	0.7	0.7
HEPES	10	10	10
BDM	7.5	7.5	7.5

Since “normal” plasma ion concentrations are species specific (UoM, 2009), we calculated a percentage change of [K^+^]_o_ and [Na^+^]_o_ from the lowest values used in our previous mouse study (George et al., 2015). Thus, [K^+^]_o_ was changed in this study by 52% (4.56–6.95 mM) and [Na^+^]_o_ by 5.4% (145.5 to 153.3mM) from historic guinea pig Tyrode's solutions (Veeraraghavan et al., 2012). By this method, we designed Solutions A and B as noted in Table 1.

Solutions were perfused at 37°C, pH 7.4. In each experiment, historic laboratory Tyrode's solution without the gap junction uncoupler CBX was perfused for 30 min at the beginning of the experiment, and then Solutions A and B were perfused for 10 min in a random order without CBX. This was followed by CBX (15 and 30 μM) in Solution A or B.

All conduction measurements were taken 10 min after the new perfusate reached the heart to control for the amount of time each heart was exposed to the solution, and because we previously demonstrated CBX slowed conduction to near steady-state values within 10 min (Veeraraghavan et al., 2015). Hearts were paced from the anterior left ventricular (LV) epicardium with a unipolar AgCl wire at basic cycle lengths (BCL) of 300 and 160 ms with a 5 ms pulse at 1.5 times pacing threshold (Veeraraghavan et al., 2013). A baseline BCL of 300 ms was chosen to mimic physiological resting guinea pig heart rate, while 160 ms BCL has been shown to decrease CV (Girouard et al., 1996; Akar et al., 2000; Lou et al., 2012).

### Transmission electron microscopy

Left ventricular tissue from each intervention reported (3 hearts per intervention, 3 samples per heart) was sectioned into 1 mm^3^ cubes. The sections were fixed in 2.5% glutaraldehyde at 4°C overnight and then transferred to PBS at 4°C. The tissue was processed as previously described (George et al., 2015). Images were collected using a transmission electron microscope (JEOL JEM1400) at 150,000 X magnification. Measurements were obtained using ImageJ (NIH) from 15 perinexi per sample. Total number of perinexi measured was 135.

### Optical mapping

The voltage sensitive dye di-4-ANEPPS (15 μM) was perfused for 10 min before the start of the experimental protocol. Cardiac motion was reduced with 2,3-butanedione monoxime (BDM, 7.5 mM). Hearts were further stabilized by applying light pressure on the posterior surface of the heart.

The dye was excited with a halogen light source (MHAB-150 W, Moritex Corporation) with an excitation filter of 510 nm (Brightline Fluorescence Filter). An emission filter of 610 nm [610FG01-50(T257), Andover Corporation] was used before the emitted light was recorded using a MiCam Ultima CMOS L-camera (SciMedia), sampling at a rate of 1 kHz. Images were captured on a 100 × 100 array with an inter-pixel resolution of 0.1 mm.

CV was calculated as previously described (George et al., 2015) using an algorithm by Bayly et al. (1998). Briefly, maximum rate of optical action potential rise at each pixel was calculated to determine activation time. A parabolic surface was fit to activation times to determine vectors for CV at each pixel. CV was quantified in two directions, longitudinal and transverse (CV_L_ and CV_T_, respectively), with anisotropic ratio (AR) calculated as CV_L_/CV_T_. Conduction in each direction was calculated from a group of vectors that were within 5 pixels and with an angle of ±8° from direction of longitudinal (fastest) or transverse (slowest) propagation. The first vectors immediately adjacent to the site of pacing were excluded to reduce pacing artifacts in the CV analysis. CV was only quantified up to 32 pixels from the site of pacing in the longitudinal direction and 16 pixels in the transverse direction to reduce the contribution of conduction breakthrough. Longitudinal CV was only quantified if there were at least 10 vectors meeting the criteria above and transverse CV was quantified if at least 50 vectors met the criteria above.

Rise time (RT) was calculated by the time difference from 20 to 80% of the peak fluorescent action potential amplitude during depolarization. RT was calculated both in the longitudinal (RT_L_) and transverse (RT_T_) directions. Action potential duration (APD) was calculated as the difference between the activation time and 85% repolarization from peak action potential amplitude.

### Western blotting

Hearts (*n* = 3), perfused with respective solutions for 30 min, were snap frozen. Tissue was homogenized in a RIPA lysis buffer (50 mM Tris pH 7.4, 150 mM NaCl, 1mM EDTA, 1% Triton X-100, 1% sodium deoxycholate, 2mM NaF, 200 μM Na3VO4) supplemented with HALT Protease and Phosphatase Inhibitor Cocktail (ThermoScientific). Following sonication and clarification by centrifugation, the BioRad DC protein assay was employed to determine and normalize protein concentrations prior to analysis. SDS-PAGE electrophoresis was performed as previously described using 4–20% NuPage Bis-Tris or 3–8% Tris-Acetate gels (Life Technologies) which were then transferred using the Trans-Blot Turbo system (BioRad) to low-fluorescence PVDF membrane and blocked with 5% BSA in TNT buffer (0.1% Tween 20, 150mM NaCl, 50mM Tris pH 8.0) for 1 h at room temperature. Membranes were then incubated with rabbit anti-phospho-Cx43^Ser368^ (1:1000 in 5% BSA TNT, #3511 Cell Signaling Technology) overnight at 4°C. Following several washes in TNT, secondary detection was performed using goat anti-rabbit HRP antibody (1:5000, abcam) for 1 h at room temperature. Bound antibody was detected post washing using Clarity Western ECL Substrate (BioRad) and imaged using the BioRad Chemidoc MP system. Membranes were then stripped with Re-Blot Plus Strong (Millipore) according to manufacturer's instructions. To detect total Cx43, stripped membranes were blocked for 1 h at room temperature in 5% milk in TNT buffer, and subsequently incubated overnight at 4°C with primary antibodies against Cx43 (1:4000, C6219 rabbit, Sigma Aldrich), and alpha-tubulin (1:4000, T6199 mouse, Sigma-Aldrich) diluted in 5% milk TNT. Membranes were then washed and incubated with the fluorescently distinct secondary antibodies goat anti-mouse AlexaFluor555 and goat anti-rabbit AlexaFlour647 (both 1:1000 in milk TNT, Life Technologies) for 1 h at room temperature. Following several washes in TNT, membranes were fixed in methanol, dried, and imaged using the Biorad Chemidoc MP System. Protein expression was quantified by densitometry using ImageLab software (BioRad), Cx43 expression levels were normalized to alpha-tubulin, and Cx43 phosphorylate Serine 368 (Cx43-p368S) to total Cx43.

### Statistics analysis

Statistical analysis was performed using a two tailed Student's *t*-test for both paired and unpaired data. A *p* ≤ 0.025 after Bonferrroni correction was considered significant. All values are reported as mean ± standard error unless otherwise noted.

## Results

### Conduction velocity—control conditions (0 μM carbenoxolone)

**Figure 1:**
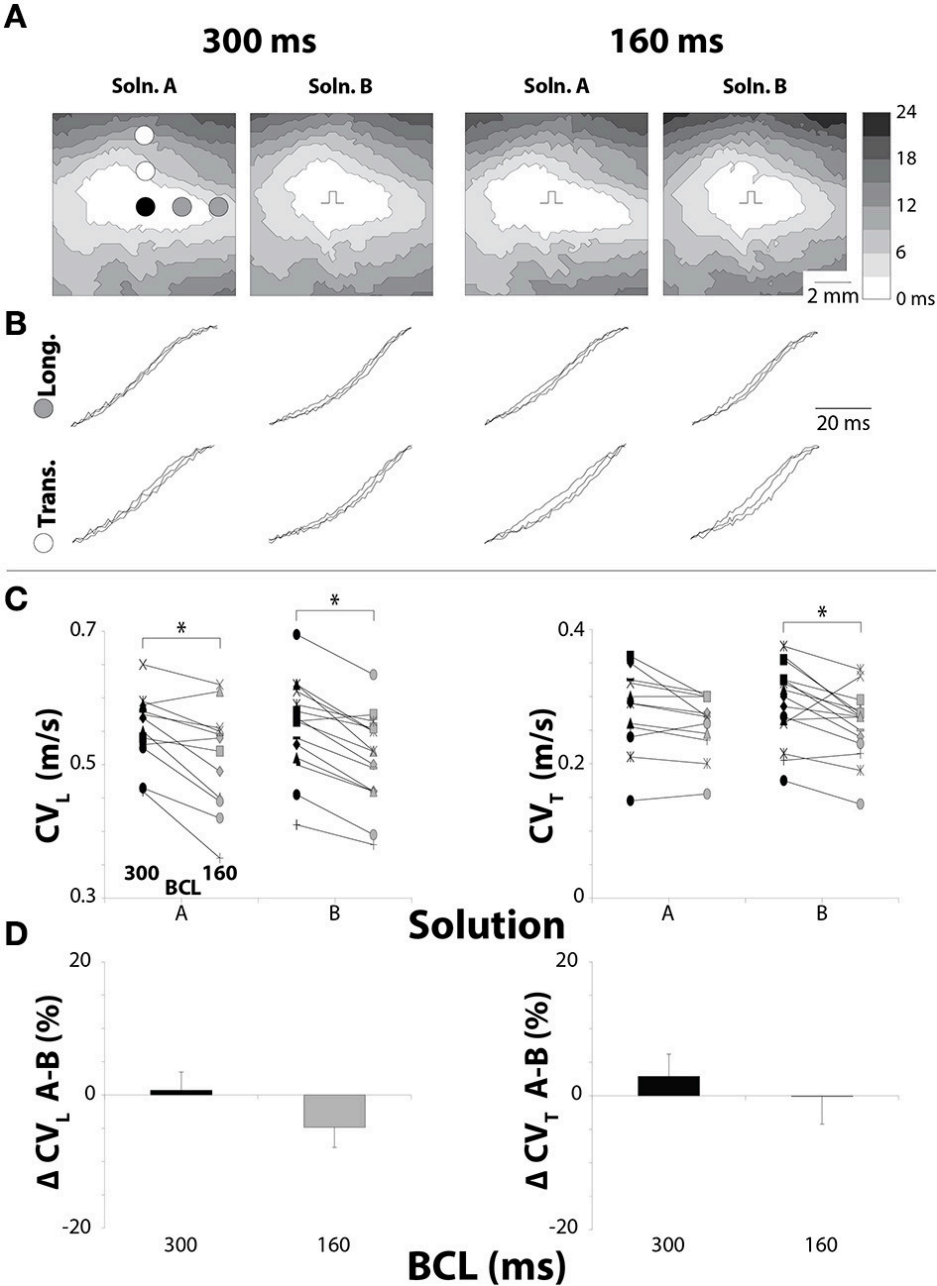
**Pacing rate but not solution composition alters CV in hearts with normal GJ coupling. (A)** Representative isochrones from hearts perfused without CBX, showing conduction slowing between pacing rates, but not solutions. **(B)** Uniformly spaced action potential upstrokes from the same hearts as pictured in **(A)**, demonstrating temporal upstroke separation as another indicator of decreased CV. **(C)** Black symbols: 300 ms BCL, Gray symbols: 160 ms BCL. CV measurements for longitudinal and transverse directions. CV decreased due to increased pacing rate for each combination except Solution A in the transverse direction. **(D)** Percent changes in CV_L_ and CV_T_ between Solutions A and B show no changes in CV due to perfusate. ^*^p < 0.025 between pacing rates.

Cardiac conduction was quantified from guinea pig ventricles to determine whether increasing [K^+^]_o_ and decreasing [Na^+^]_o_ produced similar effects in *ex vivo* guinea pig as it did in mice hearts (George et al., 2015). Representative epicardial isochrones in Figure 1A demonstrate the effect of solution composition on the spatial extent of epicardial activation at both 300 and 160 ms BCL. These maps suggest that cardiac conduction under control conditions was similar when hearts were perfused with Solution A or B. Further, representative isochrones maps suggest that reducing BCL decreases CV_L_ and CV_T_, consistent with sodium channel restitution kinetics (Allouis et al., 2006). Representative action potential upstrokes in Figure 1B from equally spaced sites demonstrate slower CV_T_ than CV_L_ as evidenced by increased temporal separation between action potential upstrokes in the transverse direction.

Reducing BCL significantly decreased CV_L_ for both Solutions A and B (Figure 1C). Interestingly, reducing BCL to 160 ms with Solution A did not decrease CV_T_, although a trend was observed (*p* = 0.03). On the other hand, changing BCL with Solution B caused a significant decrease in CV_T_. For all experiments, solution composition did not significantly change CV_L_ or CV_T_ under control conditions (Figure 1D). These data suggest that solution composition does not significantly modulate conduction dependence on pacing rate with normal GJ coupling, because both solutions changed CV similarly under all conditions.

### Conduction velocity—15 μM carbenoxolone

**Figure 2:**
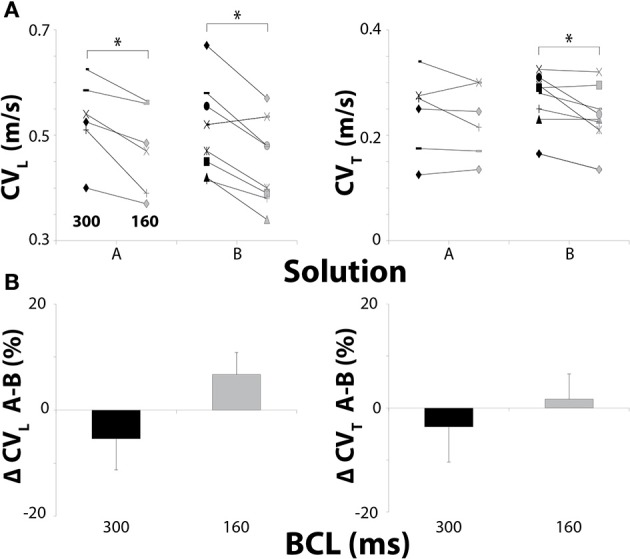
**Conduction in hearts perfused with 15 μM CBX. (A)** Black symbols: 300 ms BCL, Gray symbols: 160 ms BCL. Effects of BCL on CV_L_ and CV_T_. **(B)** Percent changes in CV_L_ and CV_T_ between Solutions A and B. Solution did not change CV at 15 μM CBX. ^*^p < 0.025 between pacing rates.

We previously demonstrated that GJ inhibition with 15μM CBX does not significantly alter CV (Veeraraghavan et al., 2012). For Solution A + 15 μM CBX, reducing BCL to 160 ms significantly decreased CV_L_ but did not significantly decrease CV_T_ (Figure 2A). Under these conditions the relationship did not trend toward significance (*p* = 0.70) in contrast to control.

Similar to control, reducing BCL from 300 to 160 ms still decreased CV_L_ and CV_T_ in hearts perfused with Solution B (Figure 2A). Importantly, comparing CV_L_ and CV_T_ for Solutions A and B did not reveal conduction differences when hearts were perfused with 15 μM CBX at either pacing rate (Figure 2B), and this is similar to findings under control conditions. These data suggest that solution composition does not significantly modulate conduction dependence on pacing rate when a GJ uncoupler does not measurably slow conduction.

### Conduction velocity—30 μM carbenoxolone

**Figure 3:**
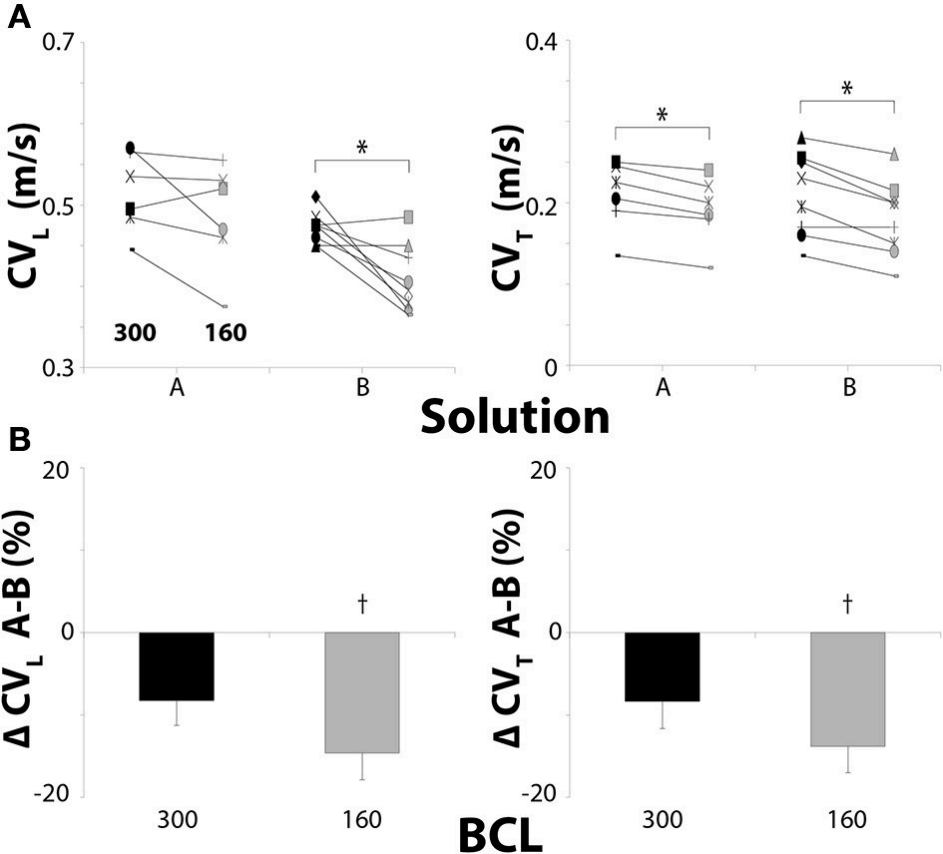
**Conduction in hearts perfused with 30μM CBX. (A)** Black symbols: 300 ms BCL, Gray symbols: 160 ms BCL. Effects of BCL on CV_L_ and CV_T_. **(B)** Percent changes in CV_L_ and CV_T_ between Solutions A and B. Solution B decreases CV_L_ and CV_T_ more than Solution A at 160 ms BCL with 30μM CBX. ^*^p < 0.025 between pacing rates. ^†^*p* < 0.025 between Solutions A and B.

Next, we increased CBX to 30 μM since it has been previously demonstrated that CBX between 20 and 100 μM can slow cardiac conduction (Spray et al., 2006). For Solution A + 30 μM CBX, reducing BCL from 300 to 160 ms did not significantly decrease CV_L_ but it did decrease CV_T_ (Figure 3A).

Similar to control and 15 μM CBX, CV_L_ and CV_T_ significantly decreased when BCL was reduced to 160 ms in hearts perfused with Solution B + 30 μM CBX (Figure 3A). Thus, rapid pacing, in hearts perfused with Solution B, decreased CV in both the longitudinal and transverse direction for all degrees of GJ uncoupling, whereas the same was not true for hearts perfused with Solution A.

Similar to conditions above, the change in CV_L_ and CV_T_ between Solutions A and B were not significant at 300 ms BCL (Figure 3B) with 30 μM CBX. However, Solution B decreased CV_L_ and CV_T_ significantly more than Solution A at 160 ms BCL (Figure 3B, †). These data demonstrate that significant GJ uncoupling can exacerbate CV dependent differences on [K^+^]_o_ and [Na^+^]_o_ when pacing rate is increased.

### Perinexal width

**Figure 4:**
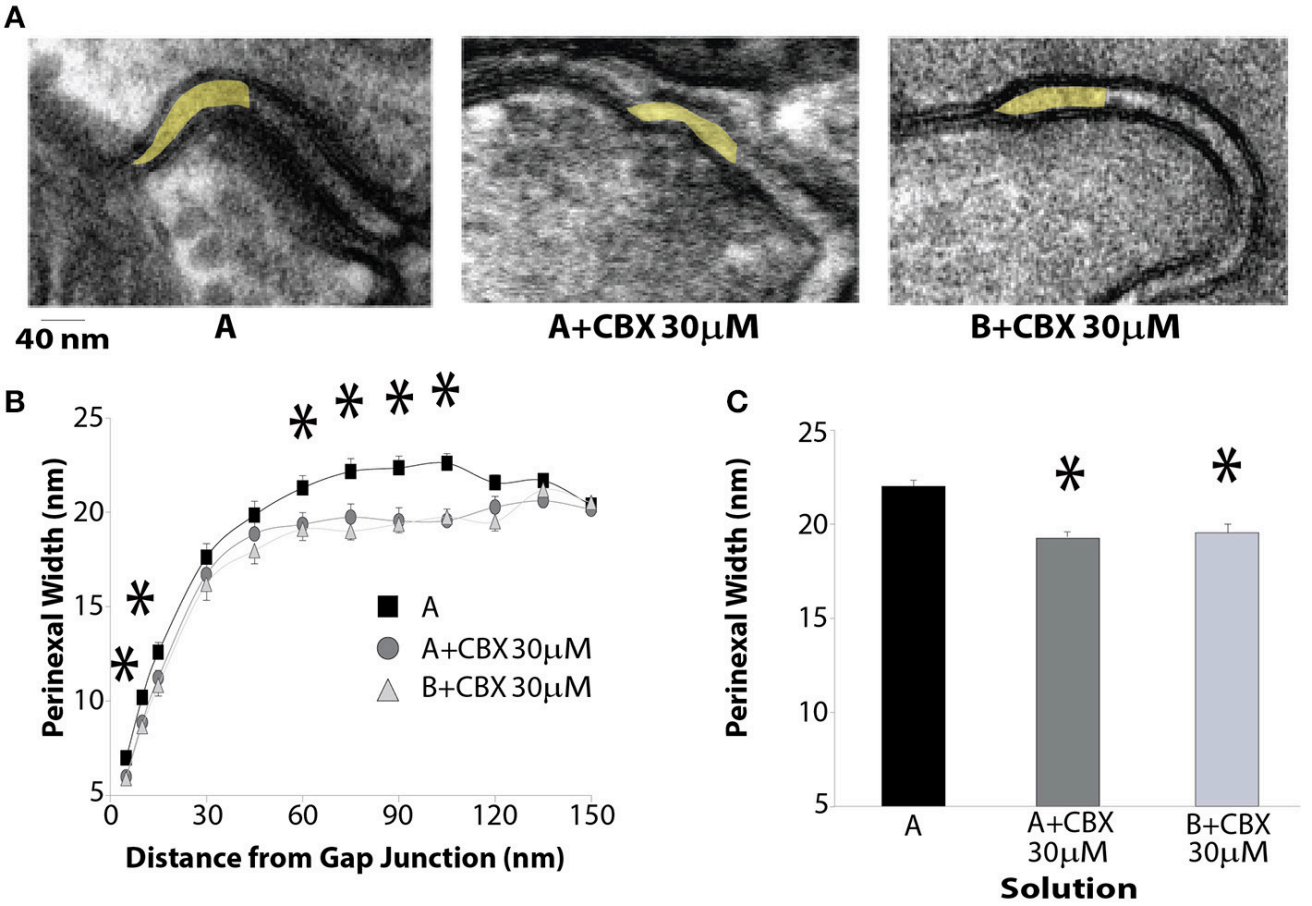
**CBX decreases perinexal width. (A)** Representative transmission electron microscopy images of the edge of a GJ plaque and the perinexus (highlighted in yellow). **(B)** Perinexal width as a function of distance from the GJ plaque. CBX decreases intercellular separation between 60 and 105 nm from the GJ. **(C)** Summary Data between 60 and 105 nm from the GJ plaque. ^*^p < 0.025 from Solution A.

The perinexus is defined as the extracellular microdomain immediately adjacent to GJ plaques, and these microdomains, rich in the cardiac isoform of the voltage gated sodium channel, have been proposed as the structural unit of a cardiac ephapse (Rhett et al., 2013; George et al., 2015; Veeraraghavan et al., 2015). Using transmission electron microscopy, the perinexus was quantified from hearts perfused with Solution A, Solution A + CBX 30 μM, and Solution B + CBX 30 μM (Figure 4A). The yellow shaded region in the images represents the first 150 nm of the perinexus adjacent to a GJ plaque. The intermembrane widths of perinexi were measured in 5 nm increments for the first 15 nm and then in 15 nm intervals up to150 nm away from the GJ (Figure 4B). Perinexal width was significantly larger with Solution A alone when compared to tissue perfused with CBX (Solution A or B). With CBX, there were no significant differences in perinexal width between Solutions A and B as summarized in Figure 4C. The data also demonstrates that the perinexal width from 60 to 105 nm is reduced by CBX independent of solution composition. Therefore, the difference in CV observed between Solutions A and B at 30μM CBX is not likely related to solution induced perinexal differences.

### Cx43 expression

**Figure 5:**
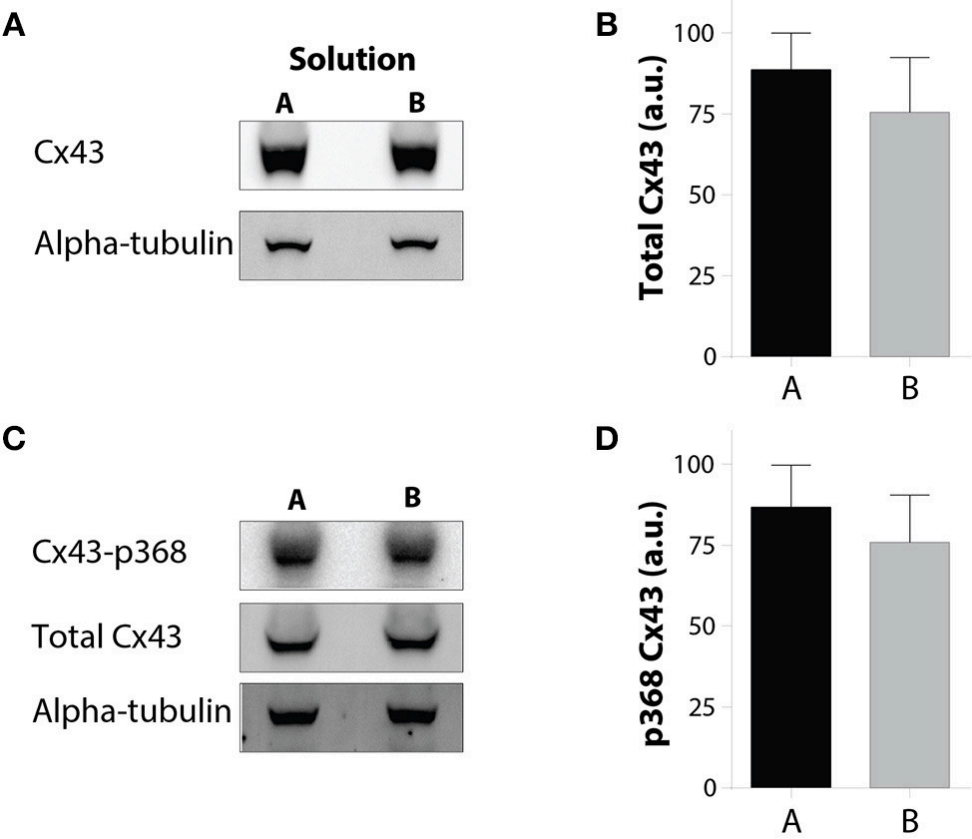
**Solution changes do not alter Cx43 expression or p368 phosphorylation. (A)** Representative Western immunoblots of Cx43 with alpha-tubulin as a loading control. **(B)** Quantification of total Cx43 expression reveals no difference between hearts perfused with Solutions A or B. **(C)** Representative Western immunoblots of Cx43-p368S and total Cx43 with alpha-tubulin as a loading control. **(D)** Quantification of Cx43-p368S expression reveals no difference between hearts perfused with Solutions A or B.

Many studies have demonstrated that CV is dependent on ionic perfusate composition (Kishida et al., 1979; Kagiyama et al., 1982; Pressler et al., 1982). We sought to determine whether the reported changes in perfusate composition can also alter Cx43 expression. Expression levels of total and Cx43-p368S were measured using alpha-tubulin as a protein loading control in hearts perfused for 30 min with Solution A or B. Figures 5A,B demonstrate that solution composition did not alter total Cx43 expression. Dephosphorylation at serine 368 leads to a cascade which internalizes Cx43 in cardiac cells (Smyth et al., 2014), and Cx43-p368S protein levels are often quantified as a correlate of non-functional Cx43 GJs (Hund et al., 2007; Palatinus et al., 2011). Importantly, Cx43-p368S expression levels were not different in preparations perfused with Solution A or B (Figures 5C,D). In a separate set of experiments, Cx43-p368S was compared to total Cx43 in hearts perfused with Solutions A or B, and freshly explanted hearts, revealing that perfusion did not alter the ratio of phosphorylated Cx43 (Supplemental Figure 1). These data suggest that solution associated conduction changes were not predominantly due to Cx43 expression changes.

### Action potential duration

**Figure 6:**
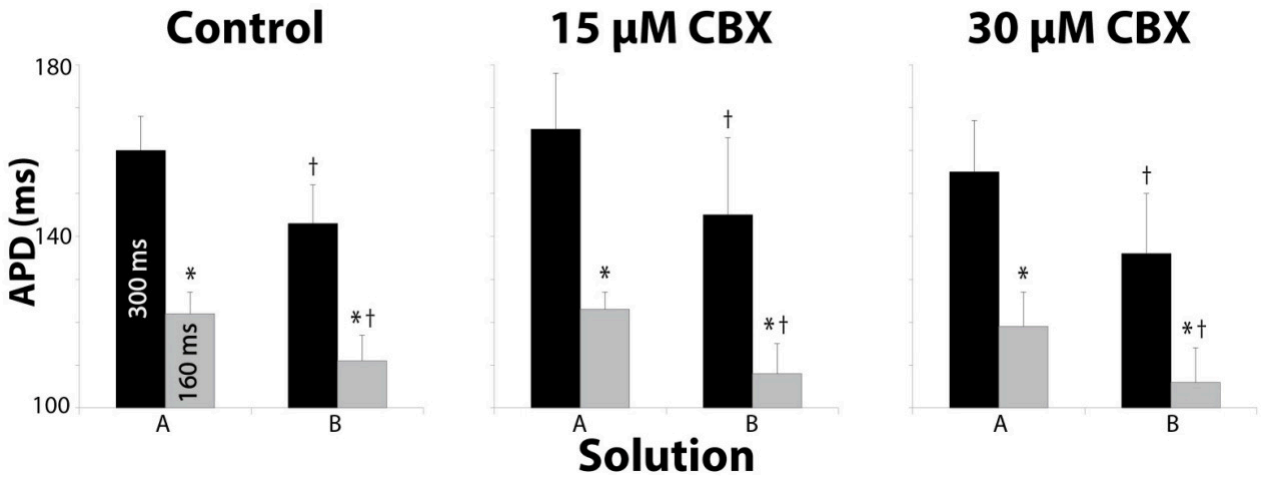
**Action potential duration measurements for all solution and pacing rate combinations tested**. Reducing BCL always shortened APD for all solutions at every CBX concentration. ^*^p < 0.025 between BCL. Solution B significantly shortened APD relative to Solution A for all CBX concentrations. ^†^*p* < 0.025 between Solutions.

APD during perfusion of Solution B was shorter than in hearts perfused with Solution A for all CBX concentrations and BCLs (Figure 6, †). As expected, APD significantly shortened with both solutions when BCL was reduced from 300 to 160ms (Figure 6, ^*^). Since APD shortened approximately equally with Solutions A and B, and the magnitude of APD shortening was similar, these data suggest that APD restitution kinetics may not explain the changes in CV reported above. In fact, one might expect that Solution A, which produced the longer APD, would decrease CV more at a 160 ms BCL, and this was not the case.

### Rise time

**Figure 7:**
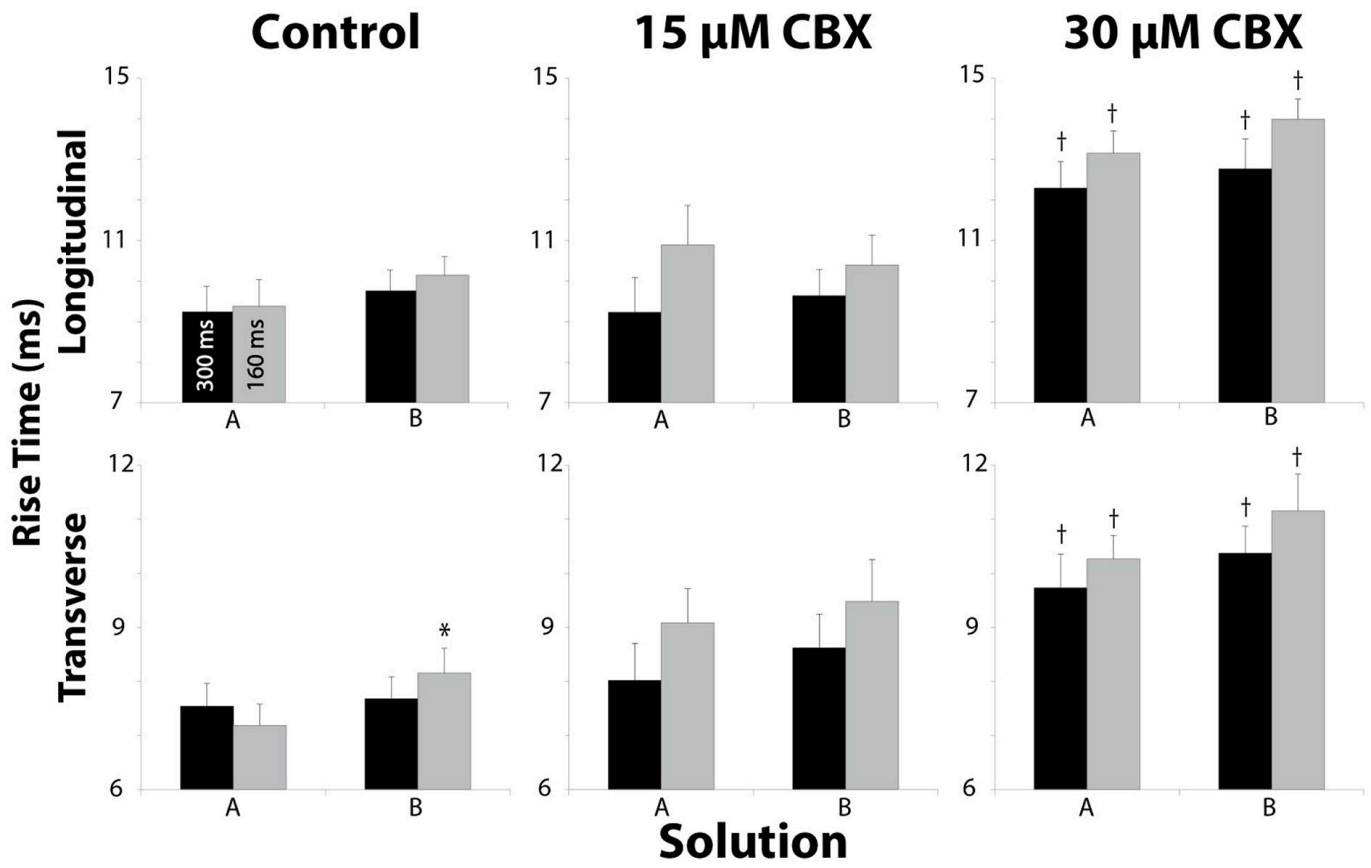
**Rise Time measurements in both longitudinal and transverse directions for all Solution and pacing rates**. Pharmacologic uncoupling with CBX progressively increased RT. ^*^p < 0.025 relative to control, ^†^*p* < 0.025 between BCL.

Optical rise time (RT) has been previously used as an inverse correlate of cellular excitability (Spach et al., 1981; Poelzing and Rosenbaum, 2004; Poelzing et al., 2004). Importantly, 15 μM CBX did not significantly alter RT relative to control (Figure 7). With 30μM CBX however, RT significantly increased (Figure 7, †). In contrast, solution composition did not significantly alter RT. Altering pacing rate also did not significantly change RT in either the longitudinal (RT_L_) or transverse (RT_T_) directions of propagation.

## Discussion

The data demonstrate how varying [K^+^]_o_, [Na^+^]_o_, and pacing rate modulates conduction sensitivity to pharmacologic GJ inhibition in guinea pig ventricular preparations. Previous studies have demonstrated that each of these factors can affect CV individually, but that small combined changes in these parameters can lead to relatively significant emergent effects on conduction (Buchanan et al., 1985; de Groot et al., 2003; George et al., 2015; Veeraraghavan et al., 2015). The present study is consistent with these finding and supports a hypothesis that small differences in artificial blood substitute solutions do not produce significant electrophysiological differences with normal GJ coupling. However, when GJ coupling is reduced, extracellular ionic composition and heart rate have important effects on cardiac conduction.

### Effects of [K^+^]_o_ and [Na^+^]_o_

The relationship between CV and [K^+^]_o_ is biphasic. Specifically, in guinea pig, it was demonstrated that CV is maximal around 8 mM [K^+^]_o_ (Kagiyama et al., 1982; Buchanan et al., 1985; Veenstra et al., 1987; Nygren and Giles, 2000). Interestingly, increasing [K^+^]_o_ from 4.6 mM in Solution A to 7.0 mM with Solution B, did not alter CV during control conditions or 15 μM CBX, which is unexpected based on previous works when [K^+^]_o_ was altered in a similar range (Kagiyama et al., 1982; Buchanan et al., 1985). One important difference between this and previous studies is that [Na^+^]_o_ is reduced in Solution B relative to A. The results show that CV is relatively insensitive to the changes in [K^+^]_o_ and [Na^+^]_o_ during normal and presumably low GJ uncoupling (15 μM CBX), with increased sensitivity at 30 μM CBX. These results are consistent with our data obtained in mice with narrow perinexal widths and presumably increased EpC (George et al., 2015).

The finding that increasing [K^+^]_o_ in guinea pig myocardium can slow conduction is consistent with the study of Pandit et al. who found that dominant rotor frequencies are decreased, suggesting slowed conduction, when [K^+^]_o_ is increased from 4 to 7 mM (Pandit et al., 2010). The intriguing finding that increasing [K^+^]_o_ in ventricular myocardium can increase CV under certain conditions and decrease it under others is an important future direction for research since increased [K^+^]_o_ is critically important to understanding electrical abnormalities in diseases such as cardiac ischemia (Kagiyama et al., 1982; Buchanan et al., 1985). Ischemia is also acutely associated with acutely increased heart rate and collapse of the extracellular space, and on a relatively longer time scale, loss of GJ coupling, (Janse and Wit, 1989; Kostin et al., 2003; Severs et al., 2004) further highlighting the importance of understanding these diverse modulators of cardiac conduction.

Increasing [K^+^]_o_ and [Na^+^]_o_ have both been associated with changes in ion channel kinetics. During hypokalemia for example, peak current density of the inward rectifier potassium current (I_K1_) is reduced, the slow component of the delayed rectifier potassium current (I_Ks_) is increased, and the rapid component of the delayed rectifier potassium current (I_Kr_) is decreased (Scamps and Carmeliet, 1989; Sanguinetti and Jurkiewicz, 1992). Additionally, co-transporters such as the Na-K pump can be activated through a combination of [Na^+^]_*i*_ and [K^+^]_o_ (Gadsby, 1984; Glitsch, 2001). Therefore, future studies will be required to elucidate additional effects of other channels, pumps, and exchangers on cardiac conduction.

### Effects of pacing rate

It is well accepted that CV decreases when pacing rate increases, due to sodium channel inactivation (Allouis et al., 2006; Stein et al., 2011). Here, we demonstrated that reducing BCL decreased CV depending on the solution composition and level of pharmacologic GJ inhibition. Specifically, reducing BCL in hearts perfused with Solution B with and without CBX decreased CV_L_ and CV_T_. The same effects were not always observed with Solution A. Our previous research suggests that EpC can support normal conduction when GJs are reduced (Veeraraghavan et al., 2012, 2015; George et al., 2015). The finding that Solution A is less sensitive to GJ uncoupling, suggests that Solution A may promote EpC by maintaining sodium channel availability (lower [K^+^]_o_) and increasing sodium driving force ([Na^+^]_o_). Further, even greater sodium channel inactivation by rapid pacing does not significantly decrease CV even with GJ uncoupling sufficient to reduce CV.

### Effects of gap junctional coupling

In the current study, increasing the concentration of CBX appeared to progressively decrease CV. This agrees with previous research which demonstrated that concentrations as high as 15 μM CBX do not significantly decrease CV, while concentrations between 20 and 100 μM CBX can decrease CV (Spray et al., 2006; Veeraraghavan et al., 2012, 2015). According to previous research, the IC50 of GJ inhibition by CBX is between 50 and 100 μM (Ye et al., 2003; Bruzzone et al., 2005; Spray et al., 2006; Suadicani et al., 2006). The observation that a significant CV decrease secondary to CBX can be modulated by solution composition and cardiac hydration further suggests that using conduction velocity to estimate IC50 of CBX may be complicated by alternative modes of conduction.

It is also possible that ionic differences in solutions altered Cx43 functional expression. However, the lack of a significant total or Cx43-p368S change in Cx43 expression suggests, but does not prove, that reported results are not due to GJ remodeling.

### Effects on rise time

Previous studies suggest that I_Na_ correlates with CV and that the maximal rate of action potential upstroke rise (dV/dt_max_) is a correlate of peak I_Na._ This is supported by studies which demonstrated that decreasing I_Na, max_ can decrease dV/dt_max_ and CV (Shaw and Rudy, 1997). Optical RT is an inverse correlate of dV/dt_max_ (Spach et al., 1981; Poelzing and Rosenbaum, 2004; Poelzing et al., 2004). The theory of cardiac conduction reserve during GJ uncoupling suggests that RT should first decrease during GJ uncoupling without a measurable effect on cardiac conduction (Rudy and Quan, 1987; Cole et al., 1988; Shaw and Rudy, 1997). We never observed such a relationship. In short, RT either did not change with GJ uncoupling by CBX or it increased, and this is consistent with other similar studies (Jalife et al., 1989; Rohr et al., 1998). However, it is important to note that CBX is a pharmacologic GJ uncoupler with off target effects, and this may explain a lack of agreement with theoretical predictions.

### Effects on perinexal width

Our group previously demonstrated that changing bulk interstitial volume can modulate CV (Veeraraghavan et al., 2012, 2015). We also demonstrated that CV is inversely correlated to perinexal width (George et al., 2015; Veeraraghavan et al., 2015). In other words, increasing perinexal width can decrease ventricular CV. To our knowledge, this is the first demonstration that CBX can decrease perinexal width, which from previous work should increase CV. An important difference in this study compared to our previous manuscripts (Veeraraghavan et al., 2012; George et al., 2015) is that changes in perinexal width in this study were caused by pharmacological intervention with CBX, instead of mannitol or altered calcium concentrations. As changes in perinexal width due to CBX have never been previously reported, it is difficult to discuss the mechanistic link between perinexal width and CBX since this was also not explored in this study. However, the relationship between CBX and perinexal width may be related to a study by Goldberg et al. demonstrating that glycyrrhetinic acid derivatives disrupt gap junction plaques (Goldberg et al., 1996), which could alter the structure of the perinexus. Therefore, these results suggest that 15 μM CBX may promote EpC while simultaneously reducing GJ coupling. By this mechanism, modest levels of CBX GJ uncoupling may not alter CV.

### Limitations

This study relies entirely upon using pacing protocols as well as pharmacological GJ inhibitors. However, the findings that altering [Na^+^]_o_ and [K^+^]_o_ can modulate cardiac conduction with pharmacologic inhibition is similar to findings in mice heterozygous null for Cx43 (George et al., 2015). It will be interesting to know in the future whether connexin targeting peptides behave similarly to glycerhetinic acid derivatives.

It is well established that the electromechanical uncoupler BDM can affect cardiac conduction, and may have therefore confounded the results here. While this possibility cannot be excluded, BDM was present for each experiment, and all electrophysiologic comparisons were paired. However, perinexal measurements were analyzed in an unpaired fashion, and BDM may have altered these results. Yet, we previously demonstrated that BDM does not change perinexal width relative to freshly excised hearts (Veeraraghavan et al., 2015).

It has been shown that Cx43 localization can change due to cardiac disease (Spragg et al., 2005; Spragg and Kass, 2006; Duffy, 2012). While we provide evidence that perinexal spacing was only changed for samples that included CBX, alteration of Cx43 localization could account for some of the electrophysiological changes that were seen. However, the effects quantified in this study occurred in 10 or less minutes and were independent of perfusion order. Therefore, it seems unlikely that Cx43 cellular expression patterns were significantly altered on this time scale.

The mechanisms that underlie this atypical conduction response to perfusate composition during GJ uncoupling could be due to either altered cellular excitability (Kléber et al., 1987a,b) around the entire myocyte or ephaptic mechanisms (Veeraraghavan et al., 2012, 2015; George et al., 2015). Importantly, the finding that conduction slowing secondary to GJ uncoupling can be exacerbated by relatively small changes in extracellular ions suggests that the mechanism governing the CV-GJ relationship is very sensitive to ionic perturbations within the physiologic range, and this warrants additional investigation.

## Conclusions

We present evidence that altering [K^+^]_o_, [Na^+^]_o_, and heart rate have important effects on GJ mediated conduction slowing. The magnitude of ionic changes, while small, were in accordance with the original hypothesis that the changes in [K^+^]_o_ and [Na^+^]_o_ would not substantially alter CV as previously demonstrated in mice with normal Cx43 expression levels, but GJ uncoupling can produce solution dependent CV changes. The implications of this study are that relatively small physiologic changes in extracellular ionic composition do not significantly perturb cardiac electrophysiology under “normal” conditions, but these same changes may have significant effects during cardiac stress as a result of GJ remodeling or increased heart rate.

## Author contributions

All authors are fully aware of the content of the manuscript and have contributed to its writing. ME and SP were responsible for the entire direction of the project. SG was responsible for the design of the solution fluids and the TEM imaging. MZ was responsible for the immunoblotting, analysis and reporting of Cx43. JS was responsible for supervising and the immunoblotting, analysis and reporting of Cx43.

## Funding

This work was supported by an R01-HL102298 awarded to SP, and a VTCRI Medical Research Scholar Award to ME.

### Conflict of interest statement

The authors declare that the research was conducted in the absence of any commercial or financial relationships that could be construed as a potential conflict of interest.
