# Supplementary material for: Heart Rate and Extracellular Sodium and Potassium Modulation of Gap Junction Mediated Conduction in Guinea Pigs
Source: Front Physiol. 2016 Feb 2;7:16. doi: 10.3389/fphys.2016.00016 (PMC4735342; doi:10.3389/fphys.2016.00016)
Supplement: Supplementary file 1 [file Image1.PDF]

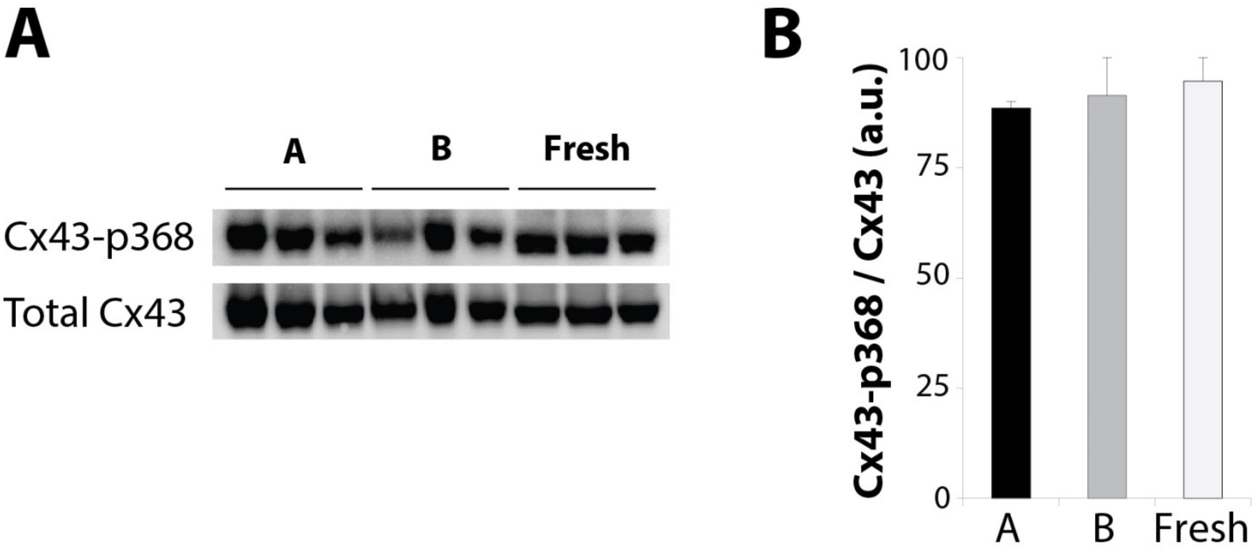

Supplemental Figure 1 – The ratio of p368 to total Cx43 in hearts perfused with Solution A or B, and freshly explanted hearts was not different. (A) Representative Western immunoblots of Cx43-p368 and total Cx43 protein expression. (B) Quantification of Cx43-p368 to total Cx43 ratio for hearts perfused with Solution A, B, and freshly explanted hearts.
